# Estimating global economic well-being with unlit settlements

**DOI:** 10.1038/s41467-022-30099-9

**Published:** 2022-05-05

**Authors:** Ian McCallum, Christopher Conrad Maximillian Kyba, Juan Carlos Laso Bayas, Elena Moltchanova, Matt Cooper, Jesus Crespo Cuaresma, Shonali Pachauri, Linda See, Olga Danylo, Inian Moorthy, Myroslava Lesiv, Kimberly Baugh, Christopher D. Elvidge, Martin Hofer, Steffen Fritz

**Affiliations:** 1 International Institute for Applied Systems Analysis, Schlossplatz 1, A-2361 Laxenburg, Austria; 2 GFZ German Research Centre for Geosciences, Telegrafenberg, 14473 Potsdam, Germany; 3 School of Mathematics & Statistics, University of Canterbury, Private Bag 4800, 8041 Christchurch, New Zealand; 4 T.H. Chan School of Public Health, Harvard University, 677 Huntington Ave, 02115 Boston, MA USA; 5 Vienna University of Economics and Business, Welthandelsplatz 1, 1020 Vienna, Austria; 6 Cooperative Institute for Research in the Environmental Sciences, University of Colorado, 216 UCB, 80309 Boulder, CO USA; 7 Earth Observation Group, Payne Institute for Public Policy, Colorado School of Mines, 1500 Illinois St., 80401 Golden, CO USA

**Keywords:** Earth and environmental sciences, Environmental social sciences

## Abstract

It is well established that nighttime radiance, measured from satellites, correlates with economic prosperity across the globe. In developing countries, areas with low levels of detected radiance generally indicate limited development – with unlit areas typically being disregarded. Here we combine satellite nighttime lights and the world settlement footprint for the year 2015 to show that 19% of the total settlement footprint of the planet had no detectable artificial radiance associated with it. The majority of unlit settlement footprints are found in Africa (39%), rising to 65% if we consider only rural settlement areas, along with numerous countries in the Middle East and Asia. Significant areas of unlit settlements are also located in some developed countries. For 49 countries spread across Africa, Asia and the Americas we are able to predict and map the wealth class obtained from ~2,400,000 geo-located households based upon the percent of unlit settlements, with an overall accuracy of 87%.

## Abstract (web summary)

Nighttime lights from satellite are combined with a map of human settlements, showing that 19% of these areas, mainly in Africa, the Middle East and Asia, have no detectable artificial light. These data were then used in models to predict well-being.

## Introduction

Despite the global poverty rate having been halved since 2000^1^, almost one billion people are still living without access to reliable and affordable electricity^2^. A lack of access to modern energy impacts health and welfare and impedes sustainable development^3–5^. Knowing the location of those one billion is crucial if aid and infrastructure are to reach them^6,7^. Traditionally, wealth and poverty have been measured through surveys of household income and consumption^8^. However, many developing countries, particularly in sub-Saharan Africa, have only rudimentary economic statistics, and in many cases, lack regional data^9–11^. The international donor community is attempting to address poverty by spending billions of dollars annually in aid for the world’s poorest countries^12^ based on the assumption that aid is flowing to where poor people live. Recent studies show, however, that aid typically does not flow to poor areas in countries, but rather to places where there is relative wealth^6,13^. Targeting resources to where the poorest live is thus crucial if extreme poverty is to be addressed^6,14^. Given the paucity of relevant information, one of the most promising methods for estimating economic activity—especially for countries with low-quality statistical systems^9,15^—is that of satellite-derived radiance^16,17^.

The Visible Infrared Imaging Radiometer Suite Day-Night Band (VIIRS DNB), a global night light satellite sensor first launched in 2012, images almost the entire Earth nightly^16^ at a resolution of approximately 750 m in the 500–900 nm spectral band^18^. Owing to its improved spatial and radiometric accuracy over its predecessor (the Defense Meteorological Satellite Program), this sensor allows for analysis of lighting at the neighborhood scale^19^. Most of the artificial light observed by VIIRS DNB comes from human settlements^20^. In addition, recent advances in the development of a World Settlement Footprint^21,22^ (WSF) mean it is now possible to estimate the amount of building infrastructure globally that has no associated satellite-detectable radiance. With an original spatial resolution of 10 m, the WSF dataset currently represents the most detailed global inventory of human settlements to date^21,22^.

Relationships between radiance and prosperity^9,23–27^ have long focused on economic predictors, for example, gross domestic product (GDP). Such proxies have proved significant in nations with measurable lighting, but for impoverished regions, where there is often little or no detectable light, these approaches are difficult to implement^9,10,28^. Studies deriving relationships between nighttime radiance and daytime imagery^10^, estimating poverty via mobile phone data^8^ or the socioecological treatment of satellite data^29^ all require upscaling and are dependent on disparate proprietary datasets^10^. Recent methods employing deep and machine learning over Africa address some of these issues, predicting asset wealth and mapping poverty using publicly available satellite imagery^30^ and spatial data^31^. However, deep learning approaches are faced with a performance-interpretability tradeoff which may inhibit adoption by the policy community^30^.

Here we demonstrate a complementary, globally scalable approach, that can spatially identify—and thus monitor—economic well-being using two publicly available global datasets on settlement footprints^21^ and radiance^20^. By combining satellite nighttime lights and the world settlement footprint for the year 2015, we show that 19% of the total settlement footprint of the planet has no detectable artificial radiance associated with it. For 49 countries spread across Africa, Asia and the Americas we are able to predict and map the wealth class obtained from ~2,400,000 geo-located households based upon the percent of unlit settlements, with an overall accuracy of 87%.

## Results

### Unlit settlements per country

**Fig. 1:**
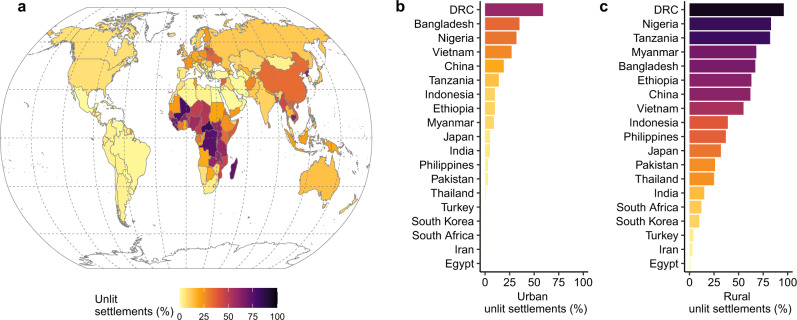
Global country-level unlit settlement percentages. **a** map of countries classified according to their percentage of settlements (building footprints) with no associated satellite-derived nighttime radiance for urban and rural regions combined. **b** African and Asian countries with population exceeding 50 million ranked according to percentage of urban unlit settlements. **c** African and Asian countries with population exceeding 50 million ranked according to percentage of rural unlit settlements.

As a first step, we determine the percentage of settlement footprints for every country that have no associated satellite-detectable nighttime radiance (Fig. 1). Globally, 19% of the planet’s settlement footprint is without detectable associated radiance, with Africa (39%) and Asia (23%) comprising the majority of this area (Supplementary Table 1, Supplementary Data, Supplementary Figs. 1, 2). If we consider just rural unlit infrastructure, the numbers rise to 65% for Africa and 40% for Asia. The large number of countries in Africa with a high percentage of building infrastructure lacking associated satellite-detected radiance is readily apparent. Several countries in the Middle East, Asia and Europe also have significant portions of unlit infrastructure. With some exceptions, North America and South America contain relatively low levels of unlit infrastructure. This can partly be explained by high levels of urbanization, particularly in South America^32^ (Supplementary Fig. 3) and high per capita energy consumption in North America^32^.

### Relationship between wealth index and unlit settlements

**Fig. 2:**
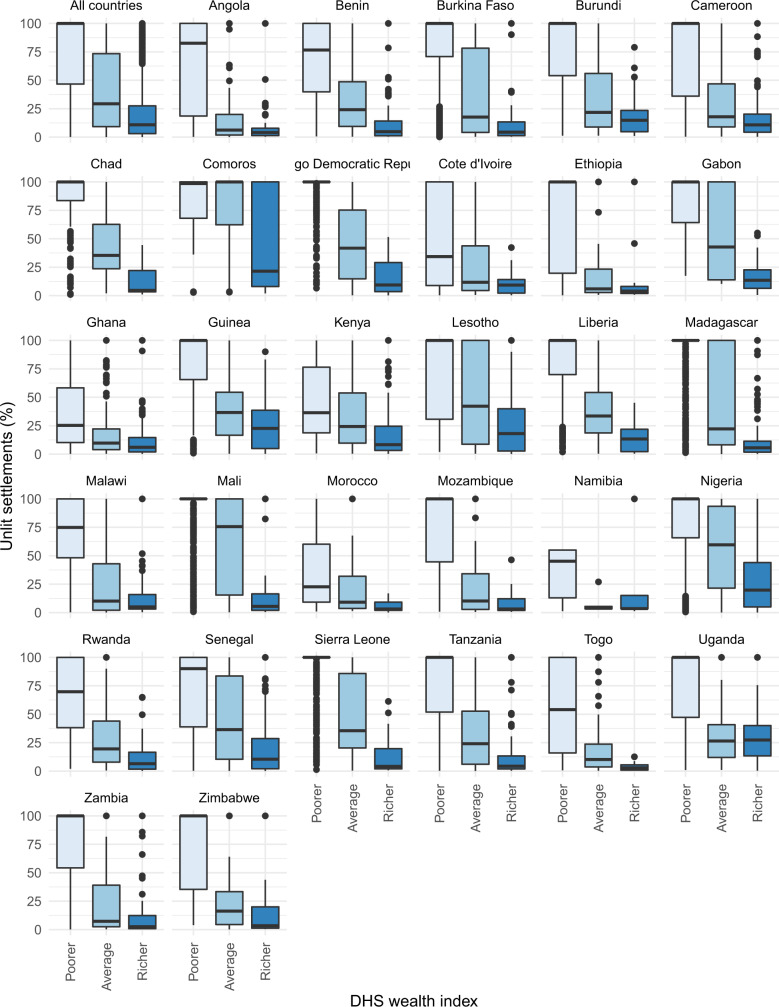
Relationships between the Demographic and Health Surveys (DHS) wealth index categories (Poorer, Average Richer) and the percentage of unlit settlements for 31 African countries. The boxplots show the mean percentage area of unlit settlements within a 2 km buffer of a DHS urban household cluster and a 5 km buffer of a DHS rural household cluster against the mode of the wealth indices of all households assigned to the household cluster. The midline represents the median with the lower and upper limits of the box being the 1st and 3rd quartiles. The lower and upper whiskers represent minima/maxima no further than 1.5 times the interquartile range from the hinge. Outliers appear as circles.

To develop our approach, we analyzed the relationship between the percentage of unlit settlements in 31 African countries (Fig. 2), 10 Asian countries and 8 countries across the Americas, with a harmonized geo-spatial wealth index calculated by the Demographic and Health Surveys (DHS) program. The DHS wealth index places individual households on a continuous scale of relative wealth from poorer to richer^33^. In total, we consider 100,602 harmonized DHS geo-located household clusters, consisting of ~2,400,000 households which have been monitored over the past three decades. In almost all countries we find a clear association between increasing percentages of unlit communities in a country and decreasing economic well-being levels.

To predict what wealth class a community is in (i.e., Poorer, Average or Richer) based upon the percentage of unlit settlements within a given pixel, we use Naïve Bayes to classify the observations, applying 10-fold cross-validation to validate the classifications. We can determine the accuracies of this prediction as the percentage of pixels classified correctly to their respective wealth classes. For the 31 African countries selected, the overall accuracy of our prediction was 93%, while for 10 Asian and 8 Americas countries it was 86% each. Overall accuracy across all surveys and countries is 86.6%, while for rural areas it is 88.2% and for urban areas 85.1% (Supplementary Tables 4–7).

### Mapping wealth classes

**Fig. 3:**
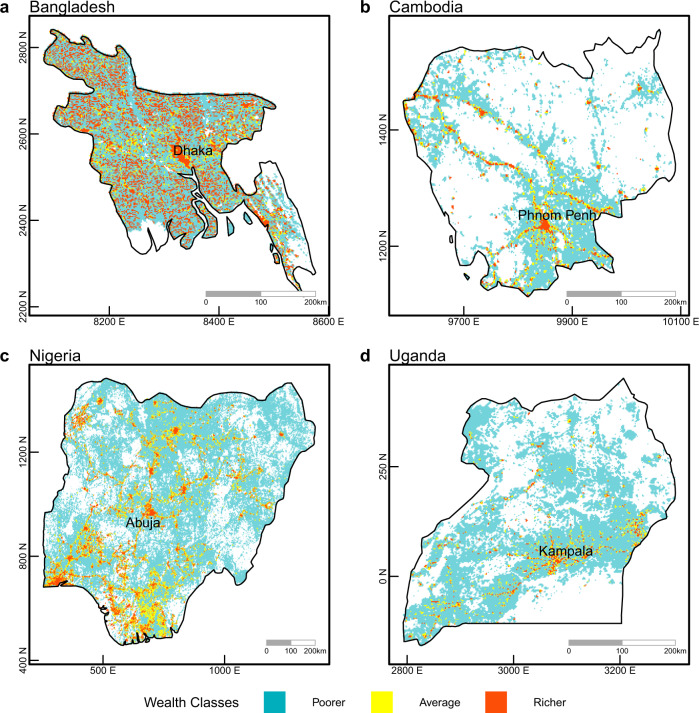
Country-level maps based on out-of-sample predictions showing the estimated relative wealth class (Poorer, Average or Richer) within a 2.5 km pixel. **a** Bangladesh. **b** Cambodia, **c** Nigeria. **d**; Uganda. Capital cities are shown. Map coordinates in kilometers north and east.

Using the results of our Naïve Bayes classification for a given country, we can apply those results to estimate the economic well-being spatially across an entire country, where only the percentage of the unlit settlements are available (Fig. 3). We show this for a set of four countries that have a significant share of unlit settlements, namely Bangladesh, Cambodia, Nigeria and Uganda. Across all four countries, large relatively poorer areas (shown in blue) are apparent, implying that those areas should be prioritized for relief. Within these poorer regions the percentage of settlement footprint coverage varies but is generally low, while the percentage of unlit settlements is variable but generally high. Yellow shaded areas imply average economic well-being. Relatively richer areas (shown in red) are typically associated with capital cities and denser urban areas.

Bangladesh experiences low to moderate levels of poorer unlit infrastructure across the entire country, while the capital Dhaka and other larger population centers have significant detectable lighting associated with settlement infrastructure. This is partly explained by the country’s large population size in 2015 (156 million) which is predominantly rural (66%)^32^ combined with widespread smallholder farms. Cambodia and Uganda contain largely poorer rural unlit settlements with only the respective capitals and a few regional centers displaying lit settlements. Cambodia had a rural population of 78% with a population of 23 million, while Uganda had a rural population of 78% with a population of 38 million^32^. In Nigeria with a population exceeding 200 million^32^ (half of which live in rural areas), we see low to moderate levels of poorer unlit infrastructure spread across the country.

### Benchmarking exercise

**Fig. 4:**
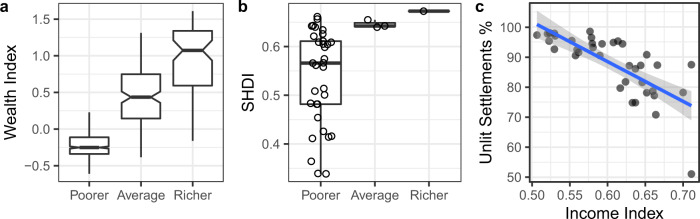
A spatial comparison of this study’s results over Nigeria. **a** Wealth index produced via deep learning^30^. **b** The Subnational Human Development Index (SHDI). **c** The share of unlit settlements predicting the SHDI Income Index. The lower and upper boxplot bounds represent 25th and 75th percentiles, respectively. The lower and upper whiskers represent minima/maxima no further than 1.5 times the interquartile range from the hinge. The notched boxplot represents the 95% confidence interval. The gray background indicates 95% confidence interval.

Finally, we performed a benchmarking exercise over Nigeria, Africa’s most populous nation, of our results against those of a recent study^30^, the Subnational Human Development Index^34^ (SHDI) and the SHDI income index (Fig. 4). Figure 4a illustrates that our results agree with a recent wealth index map produced for Nigeria employing a deep learning approach. Similarly, Fig. 4b illustrates that our wealth classes also align well with the SHDI. The SHDI is defined as an average of achievements in health, education and standard of living^34^ at the subnational level and hence provides an independent verification of our method. Figure 4c demonstrates the ability to predict the SHDI Income Index from the percent of unlit settlements.

## Discussion

Significant numbers of small rural communities and individual households in both developed and developing countries produce no satellite-detectable levels of nighttime radiance. This is due, in part, to the original resolution of the VIIRS DNB sensor (750 m) which is much coarser than an individual household, the Bidirectional Reflectance Distribution Function (BRDF) effect^35^, the fact that the satellite overpass occurs after midnight local time^16^, possible energy- or cost-saving measures occurring in communities^36^ and the increasing use of LED lighting^27^. Surface BRDF effects could be significantly different under varying illumination conditions. Nighttime lights emitted from within urban areas may exhibit a strong BRDF phenomenon, largely due to the 3D-dimensional physiognomy and structure of buildings in cities^35^. However, the correction of this effect is challenging and is an active area of research^37^.

One important limitation of currently available satellite nighttime imagery is the late satellite overpass time (1:30 am)^38^. At this time, for both developed and developing countries in rural and urban areas alike, anthropogenic light emission is likely to be reduced. Hence our method will overestimate truly unlit areas in both developed and developing countries. For developed countries, “unlit” infrastructure implies settlements with access to electricity that are either producing too little light to be measured due to their size or are intentionally reducing night light emissions by various means. In the case of developing countries, however, this will be largely restricted to small scattered rural settlements which would likely not emit enough light even at an earlier overpass, due to the use of off-grid forms of lighting^39^. Nonetheless, applying this method over time should detect changes in lighting as settlement lighting in developing countries increases and developed countries potentially decrease.

Furthermore, in rural areas, natural light sources including airglow and reflected moonlight may outshine artificial light^27,40^, with low levels of emitted radiation simply going undetected as they fall below the threshold;^20^ hence, we are likely overestimating the total number of unlit settlements globally. Conversely, very bright cities tend to cause a glow (diffuse artificial skyglow) over adjacent areas that have little or no lighting^27,41^, leading in some cases, to an overestimation of the radiance of urban and peri-urban settlements. In reality, many urban areas contain regions with varying degrees of luminosity, along with potentially poorer areas (i.e. slums) which may be only partially lit (or experience low levels of lighting). As many urban slum areas are mapped (e.g., hotosm.org) they can be monitored separately. Similarly, one brightly lit object in a pixel (e.g. a gas station) may mean the entire pixel and all structures within it are considered lit. In practice, however, adjacent glow is less likely to occur in developing countries, as even major urban centers in developing countries have far less total light emissions than wealthy countries’ urban centers^41^. To counteract some of these issues, a substantial number of steps were involved in producing the VIIRS DNB annual product including the removal of background noise, solar and lunar contamination, data degraded by cloud cover, and features unrelated to electric lighting (e.g., fires, flares, volcanoes)^20^.

While the benefit of electricity is understood, and the availability of nighttime lighting is obviously useful, there are numerous negative aspects associated with nighttime lighting. Of these, light pollution, which is the alteration of nighttime natural lighting levels by anthropogenic sources, is perhaps the best known^42^. Light pollution has global ecological consequences, poses public health issues and wastes energy and money^43^. Steps are being taken, however, to mitigate some of the negative aspects of light pollution^36,42^. In particular, our study identifies 16 % of Europe’s total settlement footprint as having no associated lighting. One partial explanation for this relatively high number of unlit settlements is a mixture of government policy and cost-saving measures by individuals and communities^36,42,44^, along with a significant rural population in Europe with many single homesteads spread across the landscape (Supplementary Fig. 3).

With an 86% average accuracy^22^, the WSF aims to capture a significant proportion of the small-scale rural settlements in addition to the comparably larger structures of cities and urban clusters^21^. It is known that information extraction proved difficult for the predecessor of the WSF, the Global Urban Footprint (GUF), when it was applied to areas of sparse and scattered settlement structures with a weak vertical expression^45^. In developing countries, the WSF thus likely underestimates the total urban footprint, with dispersed low-level single dwellings often going undetected. Furthermore, extremely poor dwellings (e.g. thatched houses) can be missed entirely^22^. As these settlements most likely lack electricity, or have only off-grid power^46^, we potentially underestimate the amount of unlit settlements in developing countries. Identifying these settlements will require additional efforts which could include crowdsourcing, in-situ data collection, earth observation techniques and more^47^. There is also a large homeless population within the urban areas of many developing nations that our methods may not be able to reflect. Future planned improvements to the WSF, along with other human settlement products^48^, could be tested using the methods proposed in our study.

In developing countries, our results are driven largely by a lack of electrification in rural areas within countries (Fig. 3, Supplementary Data). Employing the degree of urbanization approach^49^ defining rural and urban areas, we find a consistently higher level of unlit settlements in the rural domain with numerous countries experiencing greater than 80% of their rural infrastructure having no measurable associated radiance. Government agencies have prioritized expanding access for urban, rather than rural, areas, with underserved or poor areas deemed non-profitable^50^. However rural electrification holds great promise for increasing well-being, via off-grid power generation^46,50^ or grid electrification with significant positive impacts on household income, expenditure, health and education^51^. Going forward, our study could help to specifically target rural settlements in developing countries in need of electrification.

While both industry and governments are attempting to electrify developing countries^2^ and providing access to off-grid sources^46^, they will struggle to keep pace with expected population growth. In sub-Saharan Africa in particular, projections for the most optimistic scenario imply over 300 million individuals living in extreme poverty in 2030^52^. Fallout from the Covid-19 pandemic is estimated to push an additional 88 to 115 million people into extreme poverty in 2020, setting back poverty reduction by around three years^53^. The United Nations Sustainable Development Goals specifically include ‘access to affordable, reliable, sustainable and modern energy for all’^54^. This study highlights spatially the priority areas for both aid and infrastructure and could be applied to monitor progress towards the SDGs. The majority of development studies to date miss a lot of economic activity focusing exclusively on lit areas; by re-focusing on the percent of unlit infrastructure, we present a complementary approach to the estimation of economic well-being. The methods proposed here can be used to track developing countries as they electrify and developed nations as they reduce their light-energy consumption.

## Methods

### Radiance and settlement footprint data

From the Earth Observation Group (EOG) of the Colorado School of Mines, we downloaded the annual average radiance composite from the VIIRS DNB sensor (https://eogdata.mines.edu/download_dnb_composites.html; created 01.31.2017) for the year 2015^20^ (version 1). We used the version that contains cloud-free and moonlight-free average radiance values (vcm-ntl). The creation of a research quality nighttime lights product from VIIRS DNB data requires a cascading series of filtering steps to strip out data contaminated by extraneous features prior to temporal averaging^20^. We therefore consider all values remaining within the annual VIIRS DNB product after filtering as containing anthropogenic radiance. The global annual products are provided at an equal angle projection of 15 arc-seconds or approximately 500 m ground sample distance at the equator (downscaled from the 750 m original resolution). The VIIRS DNB product indicates the locations and brightness of human settlements, from large cities down to small towns and many exurban housing clusters^20^, with a nightly local equatorial overpass time of approximately 1:30 am^16^. As we are using the annual composite (i.e., a single dataset for the year 2015 made up by averaging the daily images), it will somewhat reduce the impact of the bidirectional reflectance distribution function (BRDF) effect. The stability of the average night-time lights radiance improves through the inclusion of larger numbers of observations—i.e., scan angle effects and seasonal effects (e.g., snow) are minimized^20^. The VIIRS DNB sensor is very sensitive and can capture quite low levels of lighting. As demonstrated here, a set of only 12 streetlights, built for testing purposes in an agricultural field (52.6905N, 12.4551E), away from other sources of light, is clearly visible in the VIIRS DNB data^40^.

The original WSF is provided at 10 m resolution. We obtained the resampled WSF version at 500 m^22^ reporting for each pixel the corresponding ground percent surface area covered by settlements. This maps the 2015 settlement extent, jointly exploiting over 700,000 multitemporal 2014-2015 optical Landsat-8 multispectral images and the Copernicus radar Sentinel-1 imagery^21^. It was not feasible for the WSF to consistently detect very small structures (e.g., huts, shacks, tents) because of their reduced scale, the specific building material employed (e.g., cob, mudbricks, sod, straw, fabric), their temporal nature (e.g., nomad or refugee camps), or the presence of dense vegetation preventing their identification^22^. Nonetheless, the WSF2015 is both accurate and reliable (average accuracy 86%) and outperforms similar existing datasets, having been quantitatively assessed through an unprecedented validation exercise based on 900,000 ground-truth samples^22^. While we have chosen to use the EOG (version 1) nighttime lights^20^ and WSF^22^ datasets for this study, alternative datasets exist and could be substituted in our methodology^35,48,55^.

### Definitions of buildings, settlements (rural and urban) and related radiance

Buildings are defined as covered facilities that can be used for the protection of humans, animals and things and for the production of economic goods; settlement structure is the quantitative and qualitative pattern of distribution of housing, places of work and infrastructure within a certain area^56^. With few exceptions, well-being improves going from rural areas to towns and suburbs, and then to cities^14^. The degree of urbanization identifies three types of settlements, namely: cities, with a minimum population of 50,000 inhabitants in contiguous dense grid cells (> 1,500 inhabitants per km^2^); towns and semi-dense areas, with a minimum population of 5,000 inhabitants in contiguous grid cells (>300 inhabitants per km^2^); and rural areas, consisting mostly of low-density grid cells^49^. We use the Global Human Settlement Layer (GHSL) 1 km 2015 GHS-SMOD (https://ghsl.jrc.ec.europa.eu/) to separate rural and urban areas. The GHS-SMOD delineates and classifies settlement typologies via a logic of combining a cell clusters population size, population density and built-up area densities as a refinement of the degree of urbanization method. This product is currently the state of the art in mapping rural and urban areas across the globe.

Buildings themselves are not lit per se (exceptions include e.g. façade lighting for advertising or illumination and skylights which leak light at night). Building lighting is not particularly visible from VIIRS DNB, with imaging angles strongly affecting the visibility of façade lighting^18,57^. In many cases, however, lighting associated with buildings and settlements will be due to a nearby streetlight network and e.g., lit parking lots, gas stations and more. This associated lighting can be used to imply wealth–lit parking lots imply parked cars, and settlements that are near to such lighting will likely be wealthier by association. However, the more rural the area becomes with a subsequent drop in population, the less lighting would be expected to be associated with settlements. Furthermore, as the VIIRS DNB overpass occurs after midnight, many forms of lighting are dimmed or switched off entirely at that point, meaning that settlements below a certain size will likely not be detected^22^.

### Determination of unlit WSF

We assigned the value of either lit or unlit as per the VIIRS DNB pixel (using the vcm-ntl layer, with non-anthropogenic lighting removed), to the percent of WSF infrastructure located within each VIIRS DNB pixel. Using a country dataset (https://www.naturalearthdata.com/), we could then summarize the amount of WSF area (km^2^) per country classified as being either lit or unlit. Similarly, this was then summarized at the continental level (Supplementary Table 1, Supplementary Data). Applying these results, we were able to map the global national-level unlit settlement percentages in Fig. 1 (Supplementary Figs. 1, 2).

### Development indicator data and analysis

From the World Bank’s World Development Indicators database (https://data.worldbank.org/indicator) we downloaded: GDP per capita based on purchasing power parity (PPP) (current international $) for the year 2015; electric power consumption (kWh per capita), taking the mean results between 2014 and 2016 to increase data coverage; secondary school enrollment (% gross), taking the mean results between 2014 and 2016 to increase data coverage; and urban population (% of total population) for the year 2015.

Bivariate regressions were then performed between unlit settlement footprints and each of the world development indicators by continent (Supplementary Fig. 5). Central America and the Caribbean were included in North America for this analysis. Logit transform was applied for percentages, while a log transform was used for GDP and electricity consumption. Using the model output from each of the indicators we obtained the confidence intervals for those contrasts (Supplementary Tables 2–5). We then used the above coefficients to interpret the relationships for each indicator. Preliminary analysis has shown strong correlations between the indicators. This multicollinearity makes it impossible to include them jointly in the analysis, hence we settled for a bivariate analysis.

### Household survey data preparation

The Demographic and Health Surveys (DHS) were obtained at https://dhsprogram.com/data/. The DHS collects data on a variety of factors related to household wealth, including the materials from which housing is constructed, ownership assets like televisions and bicycles and household sanitation facilities. Asset-based measures are thought to better capture households’ longer-run economic status, with the added advantage that many of the enumerated assets are directly observable to the surveyor and therefore are measured with relatively little error^10^. For all but the earliest surveys, these data have been used to create a survey-specific wealth index published by the DHS and commonly used as a correlate of a variety of health and demographic outcomes^33^. For each country we downloaded the household recode survey (i.e. wealth factor as described above with a household (HH) cluster identifier) and the HH cluster geo-coordinates (i.e. cluster centroid). Joining these two files we have a wealth factor for each household that is assigned to a cluster centroid. For comparison, an assessment was made of the World Bank’s Living Standards Measurement Study (LSMS), similar to a recent study on poverty prediction^10^, between percent unlit infrastructure and consumption for four African countries (Supplementary Fig. 4).

### Harmonizing DHS wealth data

As the DHS wealth index is survey-specific, it cannot be used for comparisons between surveys in its raw form. Thus, we constructed one all-encompassing asset index and two harmonized electricity-free asset indexes: one which has all the assets except those which require gridded electricity (e.g. excludes refrigerators) and one which excludes all assets that use any electricity whatsoever, even radios (which could be run from solar panels, batteries), etc. To do this we ran a Principal Components Analysis (PCA) across all surveys simultaneously^30^. This is similar to the method used to calculate survey-specific wealth scores, except our method generates a wealth score that is comparable across all surveys. These new wealth scores are tightly correlated with each other (across all surveys) and the original wealth scores (at the individual survey level). We run a PCA using the SVDimpute method^58^, which can impute missing records with a Singular Value Decomposition, so we did not lose records of households that did not have data regarding ownership of some possession.

### Merger of DHS and unlit WSF

From the harmonized DHS dataset, we selected only data from a survey year after 2010 and surveys that contained all eight anchor points. Furthermore, we selected only countries with data for all five of the original wealth classes. For all surveyed households in a single cluster, we took the mode of the wealth factor and assigned it to the cluster centroid. We then collapsed the original five wealth classes (i.e. poorest, poorer, average, richer and richest) into three classes, namely poorer, average and richer. To maintain anonymity, the DHS applies a random positional error. Urban clusters contain a minimum of 0 and a maximum of 2 km of error. Rural clusters contain a minimum of 0 and a maximum of 5 km of positional error with a further 1% of the rural clusters displaced by a minimum of 0 and a maximum of 10 km. We therefore applied a 2 km buffer around urban clusters and a 5 km buffer around rural clusters, assigning the amount of unlit infrastructure to those cluster centroids. Boxplots were then produced from the resulting dataset (Fig. 2). Additionally, we produced similar boxplots for Africa, Asia and the Americas split by rural and urban households to show the impact of location (Supplementary Figs. 7–9).

### Prediction

To determine the ability to use the percentage of unlit settlements to distinguish between wealth classes (i.e. poorer, average, richer), we use Naïve Bayes to classify the observations based on the Bayes formula

1 $${{\Pr }}\left({Class}\,C\right)=\frac{{Pr }\left(x{{{{{\rm{|}}}}}}{Class}\,C\right){Pr }({Class}\,C)}{{\sum }_{c}{Pr }\left(x|{Class}\,c\right){Pr }({Class}\,c)}$$

We needed probabilities for classes that can be obtained from the data as well as Pr(x|c), which we obtained by discretizing *x* and evaluating the contingency table from the data. A small correction was added to all counts to avoid division by 0. We then evaluated the accuracy using 10-fold cross-validation. We reported the resulting accuracies in Supplementary Tables 6–9. The category specific accuracy is defined as the percentage of pixels belonging to the particular category (i.e. wealth class), classified correctly to that category.

### Mapping well-being

In order to map our predicted wealth classes across entire countries, we applied the Naïve Bayes classifier to the observed DHS locations and their respective unlit settlement data within a country. We were then able to determine, at those locations, the likelihood of a wealth class being either poorer, average or richer for any given percentage of unlit settlements. Naïve Bayes produces a vector of probabilities for each pixel belonging to a particular wealth class. The pixel is then classified into the category with the highest probability. The probabilities for the most likely class thus reflect the confidence of the estimation. Selecting the most likely wealth class for each increment of unlit settlements, we then mapped the resulting estimations (Fig. 3).

### Benchmarking

Firstly we compared our resulting map of economic well-being for Nigeria with a satellite-based map of wealth estimates derived from a deep learning approach^30^. Within each level 2 district (775 in total) from gadm.org for Nigeria, we determined the mean wealth index from the deep learning approach and the median wealth class from our results, plotting these with notched boxplots. Secondly, we obtained the SHDI spatial dataset from https://globaldatalab.org/shdi/shdi/ for the year 2015. For each subnational district (37 in total), we determined the median wealth class from our results, plotting these with boxplots and the respective SHDI data points. Thirdly, we obtained the income index from the SHDI and for each subnational SHDI district determined the mean percentage of unlit settlements. We then illustrate how the share of unlit settlements predicts income while controlling for nighttime lights.

## Supplementary information

Supplementary Information

Description of Additional Supplementary Files

Dataset 1

## Data Availability

Open data were used exclusively in this study and are described under methods. The resulting national well-being maps have been deposited at Zenodo^59^. The national unlit settlement data generated in this study are provided in the Supplementary Data file.

## Code availability

All code used to produce the necessary data and figures in this study are deposited at Zenodo^59^.
