# Supplementary material for: Estimating global economic well-being with unlit settlements
Source: Nat Commun. 2022 May 5;13:2459. doi: 10.1038/s41467-022-30099-9 (PMC9072384; doi:10.1038/s41467-022-30099-9)
Supplement: Supplementary file 1 — Supplementary Information [file 41467_2022_30099_MOESM1_ESM.pdf]

Estimating global economic well-being with unlit settlements

Ian McCallum [1]
Christopher Conrad Maximillian Kyba [2]
Juan Carlos Laso Bayas [1]
Elena Moltchanova [3]
Matt Cooper [4]
Jesus Crespo Cuaresma [5, 1]
Shonali Pachauri [1]
Linda See [1]
Olga Danylo [1]
Inian Moorthy [1]
Myroslava Lesiv [1]
Kimberly Baugh [6]
Christopher D. Elvidge [7]
Martin Hofer [1]
Steffen Fritz [1]

[1] International Institute for Applied Systems Analysis, Schlossplatz 1, A-2361, Laxenburg, Austria
[2] GFZ, German Research Centre for Geosciences, Telegrafenberg, 14473, Potsdam, Germany
[3] School of Mathematics & Statistics, University of Canterbury, Private Bag 4800, 8041, Christchurch, New Zealand
[4] T.H. Chan School of Public Health, Harvard University, 677 Huntington Ave, 02115, Boston, MA, USA
[5] Vienna University of Economics and Business, Welthandelsplatz 1, 1020, Vienna, Austria
[6] Cooperative Institute for Research in the Environmental Sciences, University of Colorado, 216 UCB, 80309 Boulder, CO, USA
[7] Earth Observation Group, Payne Institute for Public Policy, Colorado School of Mines, 1500 Illinois St., 80401, Golden, CO, USA

Supplementary Information

Continental and national statistics

As a first step, we analyzed the continental statistics for unlit settlements. The following table (Supplementary Table 1) provides, by continent, the total area of all human World Settlement Footprint (WSF) settlements (km^2), the total area of unlit WSF settlements (km^2) the percentage of unlit WSF settlements, the percentage of unlit urban settlements and the percentage of unlit rural settlements. See methods for details on how these were calculated.

Supplementary Table 1. Continental statistics of Total WSF settlement area (km^2), Unlit WSF settlement area (km^2), Total Percent Unlit (%), Urban Unlit (%) and Rural Unlit (%).

Continent	Total_WSF (km2)	Unlit_WSF (km2)	Total Percent Unlit (%)	Urban Unlit (%)	Rural Unlit (%)
Africa	113923	44219	39	14	65
Asia	593547	137584	23	13	40
Europe	290257	47876	16	3	27
North America	202057	12031	6	0	15
Oceania	15733	2179	14	0	33
South America	57024	1202	2	0	8

Likewise, for each country, we summarized the total area of human WSF settlements (km^2), the total area of unlit WSF settlements (km^2), the total percentage of the unlit settlements, the percent of urban unlit settlements and the percent of rural unlit settlements (Supplementary Data). These data were used to produce Figure 1 a and b in the main document. Supplementary

Figs. 1-2 below demonstrate the global country-level unlit settlement percentages for urban and rural areas respectively. See methods for details on how these were calculated.

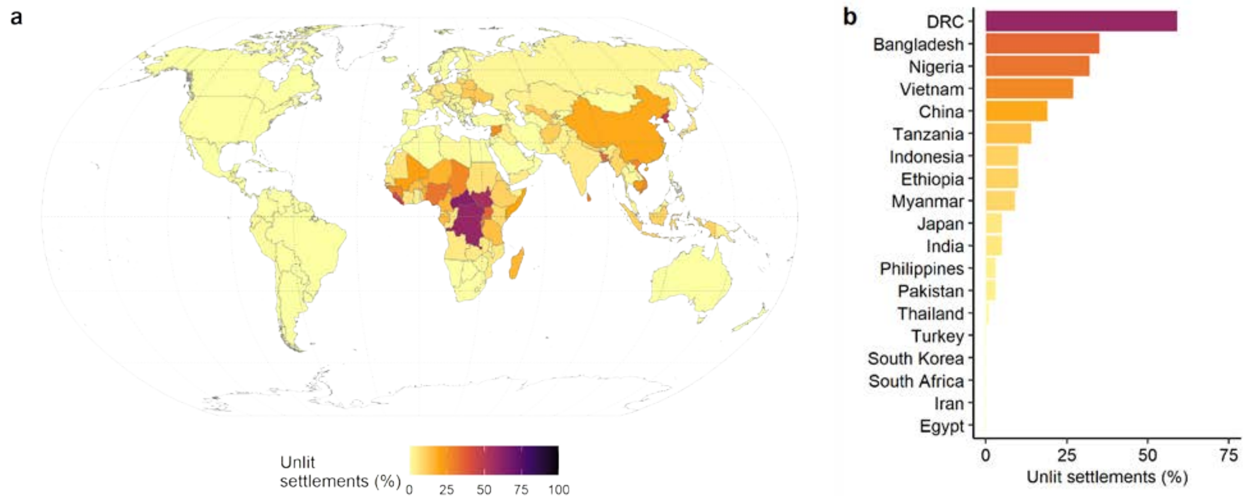

Supplementary Fig. 1. Global country-level unlit settlement percentages for urban areas. a) map of countries classified according to their percentage of settlements (building footprints) with no associated satellite-derived nighttime radiance in urban areas; b) African and Asian countries with population exceeding 50 million ranked according to percentage of urban unlit settlements.

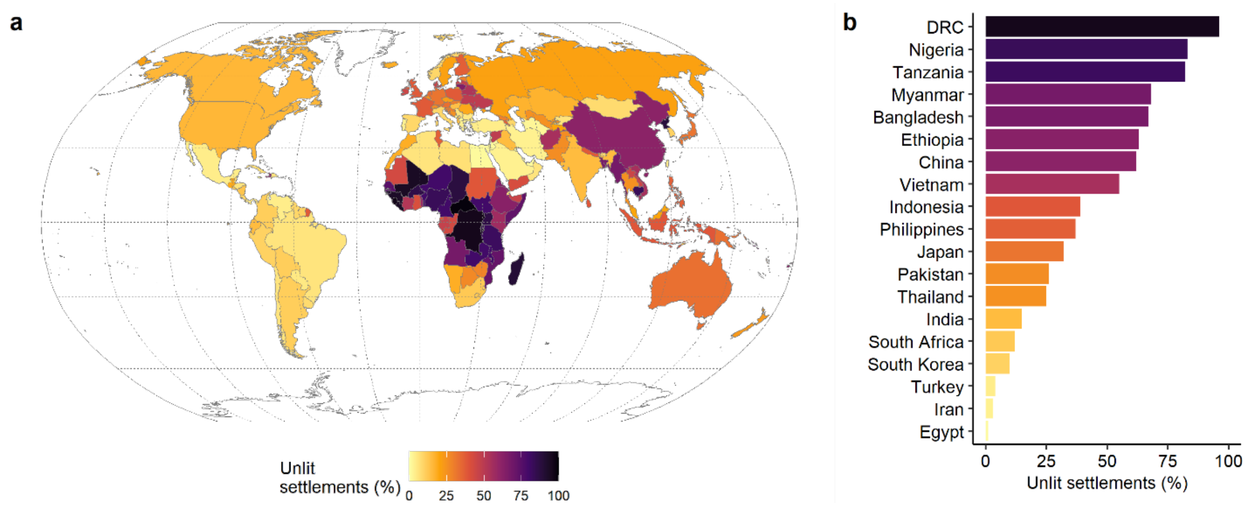

Supplementary Fig. 2. Global country-level unlit settlement percentages for rural areas. a) map of countries classified according to their percentage of settlements (building footprints) with no associated satellite-derived nighttime radiance in rural areas; b) African and Asian countries with population exceeding 50 million ranked according to percentage of rural unlit settlements.

Urban Population

One of the factors playing a potential role in the amount of unlit human settlements within a country is the percentage of the total population that is urban (Supplementary Fig. 3). Clearly visible here are the high rates of urbanization in most of South America which might partially explain the low rates of unlit infrastructure there.

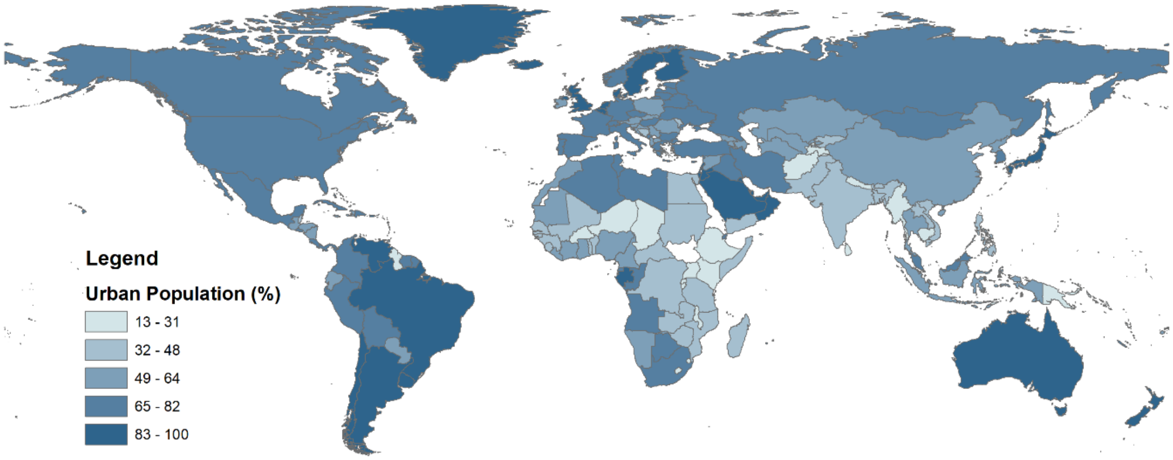

Supplementary Fig. 3. Urban population (% of total population). Source: https://data.worldbank.org/indicator

Consumption and Unlit Settlements

The World Bank's Living Standards Measurement Study (LSMS) allows for the computation of consumption spending from geocoded household surveys. Similar to a recent study on poverty prediction[1], we have generated a comparison between percent unlit infrastructure and consumption for four African countries (Supplementary Fig. 4). The majority of the rural household clusters experience high levels of unlit settlements and low levels of consumption. Urban households tend to experience higher levels of consumption and lower levels of unlit settlements, while generally remaining above the extreme poverty line. Nonetheless, some urban households are found to be associated with higher levels of unlit settlements, possibly pointing to urban slum areas recorded during surveys.

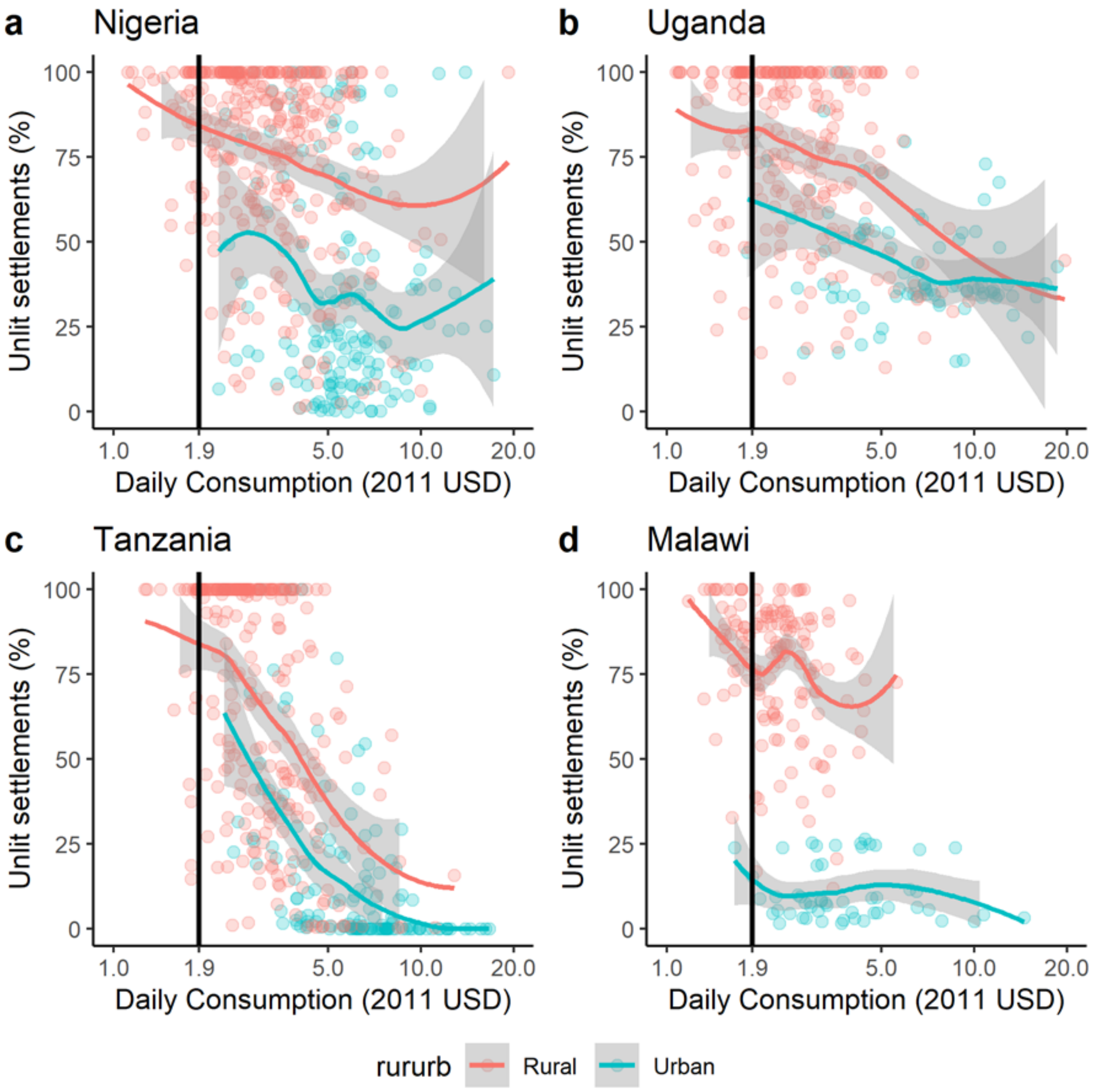

Supplementary Fig. 4. Relationship between daily per capita consumption expenditure (measured in 2011 U.S. dollars) and unlit settlements (%) at the cluster level for four African countries (a-d), based on LSMS household surveys. Vertical black lines show the official international extreme poverty line ($1.90 per person per day), and colored lines (red, blue) are fits to the data points for rural and urban clusters with corresponding 95% confidence intervals in grey.

Bivariate regressions

Comparable to studies with established relationships between economic indicators and radiance[2–7], we find significant relationships by continent between unlit settlement footprint fraction and GDP (per capita), along with electricity consumption (per capita), secondary school enrollment (% gross) and urban population (%) (Supplementary Fig. 5). Bivariate regressions (log-log) were used to model relationships within continents, treating each country as a unique observation (see Methods). In particular, the relationships are found to be statistically significant for Africa and Asia across all four indicators with large effect sizes, but less so for other continents (Supplementary Table 3).

For Africa and Asia, a 10% increase in the GDP per capita was associated with an average 10% and 9% decrease in the odds of the area being unlit, respectively. A 10% increase in per capita electricity consumption was associated with, on average, a 6% and 7% decrease in the odds of the area being unlit, for Africa and Asia, respectively. An increase of 10% in the odds of school enrollment was associated with, on average, a 3% and 2% decrease in the odds of the area being unlit, while an extra 10% in the odds of the population being urban was associated with, on average, a 4% and 3% decrease in the odds of the area being unlit, for Africa and Asia, respectively. Across all economic indicators, Africa and Asia have notably a higher share of unlit settlements than other continents, along with generally lower levels of GDP per capita, electricity consumption, school enrollment and urban population.

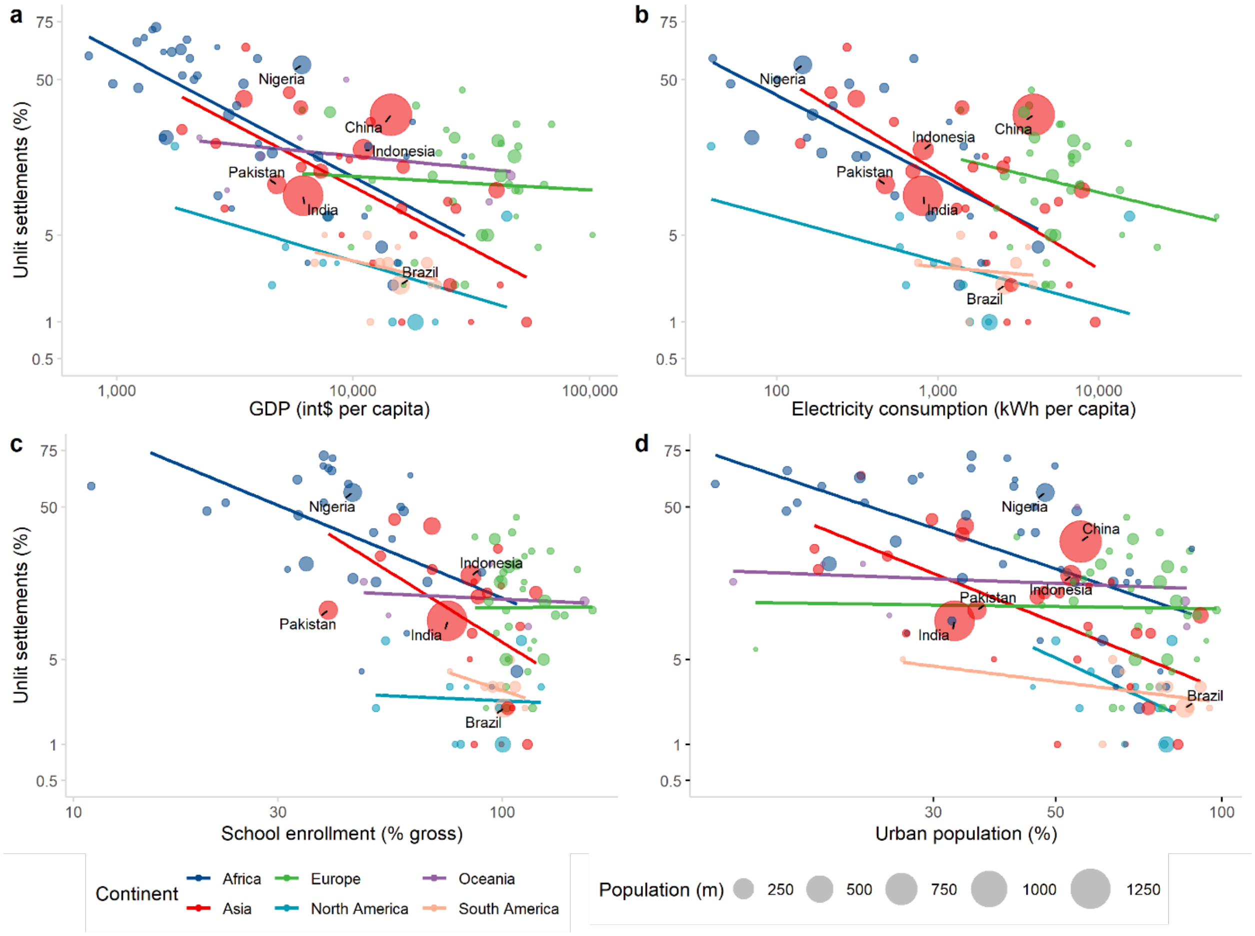

Supplementary Fig. 5. Observed trends between unlit settlement footprints and world development indicators by continent, namely, a) GDP per capita; b) electricity consumption (kWh per capita); c) school enrollment (% gross); and d) urban population (%). Oceania was excluded from 2b) having only two data points. School enrollment 2c) may exceed 100% due to over enrollment. Colors refer to continents with dot area proportional to population size (millions). Lines represent modeled trends (bivariate regressions log-log). Countries with more than 200 million inhabitants are labeled. Logit transformations are applied for school enrollment and urban population, whereas log transformations for GDP and electricity consumption.

Bivariate regressions (log-log) were used to model relationships by continent between the unlit settlement footprint fraction and GDP (per capita), electricity consumption (per capita), school enrollment (%) and urban population (%), with results of bivariate regressions by continent and indicator provided in Supplementary Tables 2-5.

Supplementary Table 2. The effects of a 10% increase in GDP (per capita) on the odds of an area being unlit (%).

Continent	Estimate	95% CI	p-value
**Africa**	-9.81	(-12.55; -6.99)	<0.0001
**Asia**	-9.25	(-12.75; -5.62)	<0.0001
**Europe**	-1.07	(-5.9; 4.01)	0.6755
**North America**	-5.43	(-11.5; 1.05)	0.1012
**Oceania**	-1.81	(-7.8; 4.57)	0.5705
**South America**	-4.05	(-17.39; 11.45)	0.5896

Supplementary Table 3. The effects of a 10% increase in electricity consumption (per capita) on the odds of an area being unlit (%). As Oceania contained only two data points, its results can be ignored.

Continent	Estimate	95% CI	p-value
**Africa**	-6.27	(-9.12; -3.33)	0.0001
**Asia**	-7.33	(-10.25; -4.33)	<0.0001
**Europe**	-2.97	(-7.39; 1.65)	0.2064
**North America**	-3.4	(-7.23; 0.6)	0.0977
**Oceania**	55.02	(-85.98; 1614.65)	0.7215
**South America**	-1.1	(-11.47; 10.48)	0.8448

Supplementary Table 4. The effects of a 10% increase in the odds of school enrollment (secondary) on the odds of an area being unlit (%).

Continent	Estimate	95% CI	p-value
**Africa**	-3.05	(-4.66; -1.41)	0.0006
**Asia**	-1.25	(-2.64; 0.15)	0.085
**Europe**	0.54	(-2.8; 3.99)	0.7567
**North America**	-0.6	(-3.19; 2.07)	0.6599
**Oceania**	0.96	(-2.97; 5.06)	0.638
**South America**	-0.34	(-3.87; 3.33)	0.8553

Supplementary Table 5. The effects of a 10% increase in the odds of an urban population on the odds of an area being unlit (%).

Continent	Estimate	95% CI	p-value
**Africa**	-3.69	(-5.12; -2.24)	<0.0001
**Asia**	-3.52	(-5.17; -1.85)	0.0001
**Europe**	-0.37	(-1.9; 1.18)	0.6363
**North America**	-2.9	(-7.09; 1.48)	0.194
**Oceania**	-0.6	(-2.85; 1.69)	0.6039
**South America**	-0.74	(-3.23; 1.81)	0.5657

Demographic and Health Surveys

The relationship between the percentage of unlit settlements and a harmonized geo-spatial wealth index (DHS) was used to develop our approach. The DHS locations we used are shown in Supplementary Fig. 6.

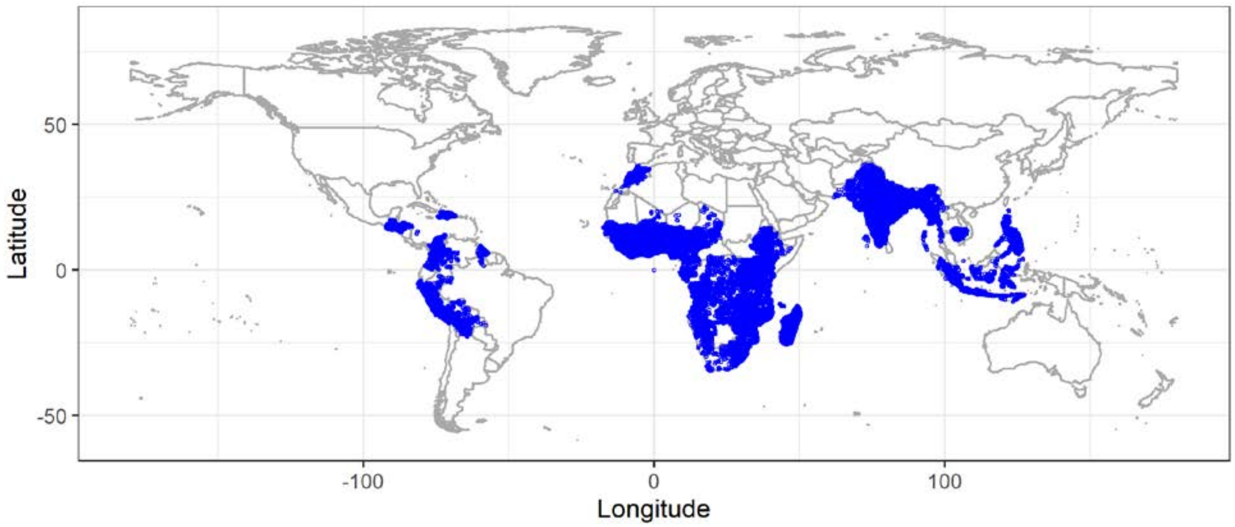

Supplementary Fig. 6. Locations of 100,602 harmonized DHS geo-located villages (blue dots) used in this study. A total of ~2,400,000 households were used as input to create the village-level data across 51 countries.

Category-specific accuracies were determined for each country using Naïve Bayes with 10-fold cross-validation. The results by continent and country are presented in Supplementary Tables 6-8 for Africa, Asia and the Americas respectively. We present results by aggregated wealth class, along with an overall value for each country. Supplementary Figs. 7, 8 and 9 present boxplots of the country-level results for Africa, Asia and the Americas, split by urban and rural classification, respectively. Supplementary Table 9 presents the overall accuracy by continent for rural and urban areas.

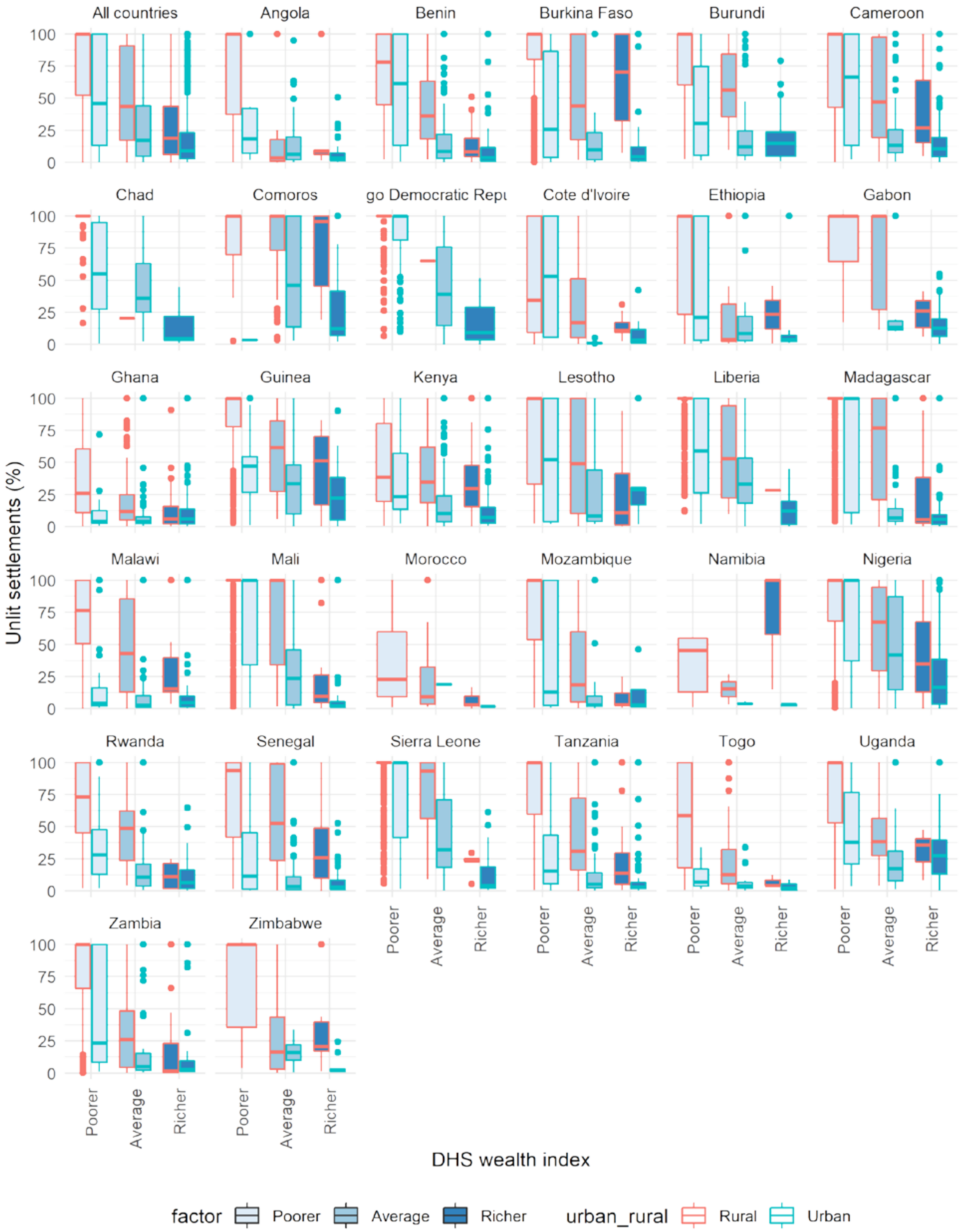

All countries
Angola
Benin
Burkina Faso
Burundi
Cameroon
Chad
Comoros
go Democratic Repu
Cote d'Ivoire
Ethiopia
Gabon
Ghana
Guinea
Kenya
Lesotho
Liberia
Madagascar
Malawi
Mali
Morocco
Mozambique
Namibia
Nigeria
Rwanda
Senegal
Sierra Leone
Tanzania
Togo
Uganda
Zambia
Zimbabwe
Unlit settlements (%)
0
25
50
75
100
Poorer
Average
Richer
DHS wealth index
factor
Poorer
Average
Richer
urban_rural
Rural
Urban

188

Supplementary Fig. 7. Boxplots for African countries showing the mean percentage area of unlit settlements within a 2 km buffer (urban) or 5 km buffer (rural) of a DHS household cluster against the mode of the wealth indices of all households assigned to the household cluster. The midline represents the median with the lower and upper limits of the box being the 1st and 3rd quartiles.

Supplementary Table 6. Category-specific accuracy by country for Africa.

	Poorer	Average	Richer	Overall
Angola	99	91.2	89.6	95.5
Benin	99.4	68.6	73.8	95
Burkina Faso	98.2	84.6	88.7	93.9
Burundi	98.4	76.8	89.2	94.9
Cameroon	100	90	100	98.9
Chad	99.3	78.6	100	95.9
Comoros	96.6	78	90.3	90
Congo Democratic Republic	99	81.6	73.6	96.7
Cote d'Ivoire	100	53.9	99.3	94.7
Ethiopia	99.2	87.3	93.9	97
Gabon	95.3	83.9	82.1	90.7
Ghana	50	96.9	70.4	81.4
Guinea	98.4	85.2	84.6	94.8
Kenya	71.2	71.5	90.9	72.6
Lesotho	100	90	100	96.9
Liberia	99.3	65.5	92.5	93
Madagascar	99.9	52.9	87	92.5
Malawi	98.8	72.6	73.3	96.1
Mali	99.4	76.6	91.7	96.6
Morocco	96	73.6	81.9	84.7
Mozambique	100	100	100	100
Namibia	98.7	83.6	86.1	96.3

Nigeria	99.5	74.9	93.6	93
Rwanda	96.5	68.7	83.2	87.2
Senegal	50	100	92.3	89.3
Sierra Leone	98.5	79.6	85.7	94
Tanzania	98.4	74.6	79.4	93.4
Togo	97.9	78.3	88.8	94.6
Uganda	100	85.7	97.6	94.6
Zambia	99.1	85.7	81.9	96.9
Zimbabwe	100	76	75	84.1
All	94.8	79.8	87.4	92.6

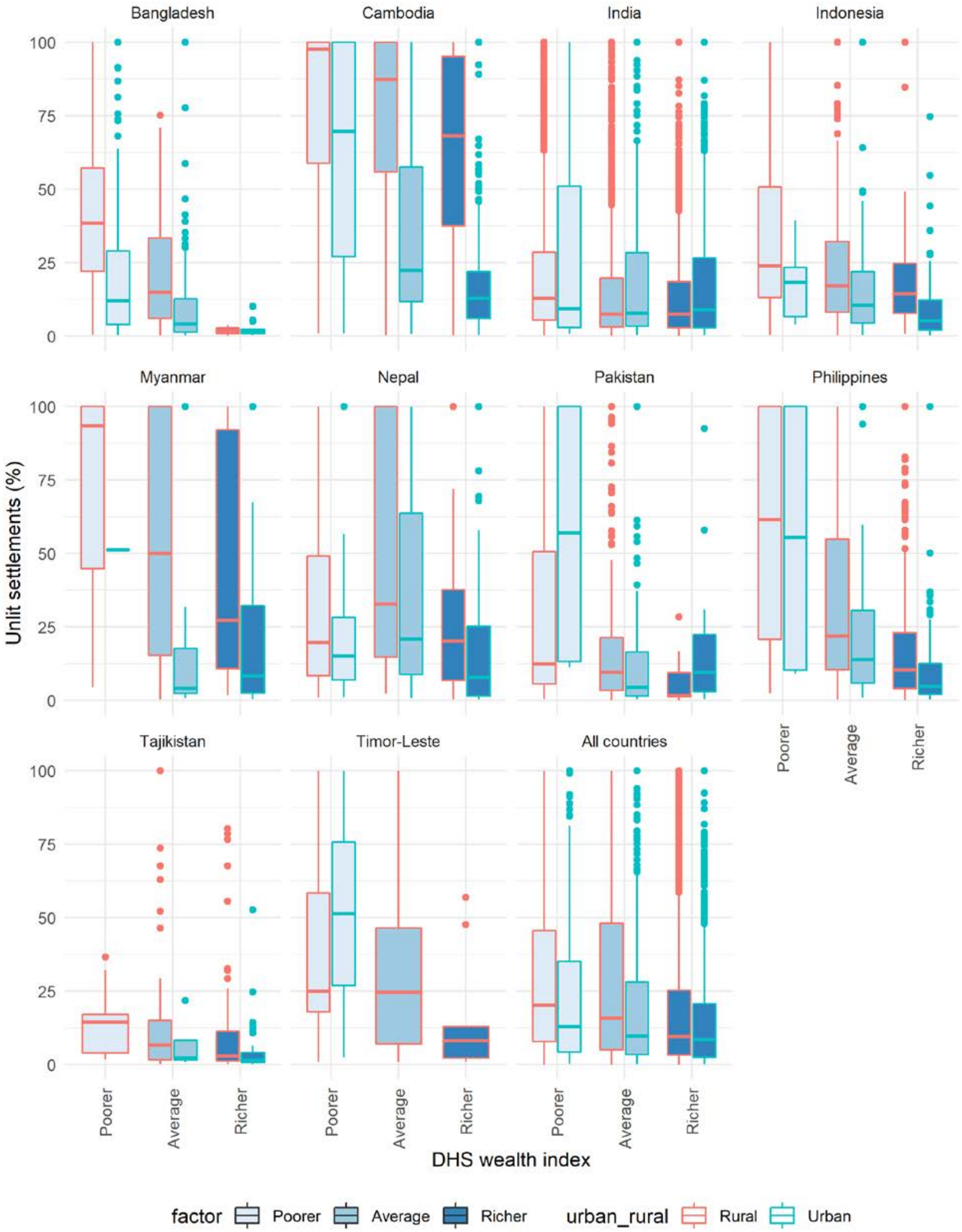
Bangladesh
Cambodia
India
Indonesia
Myanmar
Nepal
Pakistan
Philippines
Tajikistan
Timor-Leste
All countries
Unlit settlements (%)
0
25
50
75
100
Poorer
Average
Richer
DHS wealth index
factor
Poorer
Average
Richer
urban_rural
Rural
Urban

Supplementary Fig. 8. Boxplots for Asian countries showing the mean percentage area of unlit settlements within a 2 km buffer (urban) or 5 km buffer (rural) of a DHS household cluster against the mode of the wealth indices of all households assigned to the household cluster. The midline represents the median with the lower and upper limits of the box being the 1st and 3rd quartiles.

Supplementary Table 7. Category-specific accuracy by country for Asia.

	Poorer	Average	Richer	Overall
Bangladesh	98.5	76.9	61.9	96.5
Cambodia	78.6	38.1	49.9	62
India	94.6	92.1	85.6	91.2
Indonesia	46.4	94.3	81.5	77.4
Myanmar	57.1	100	80	87.7
Nepal	97.3	70.6	88.5	89.8
Pakistan	51.6	91.2	85.8	85.7
Philippines	96	95.1	69	94.2
Tajikistan	90.9	90.1	89.5	89.8
Timor-Leste	97.8	58.7	100	88.2
All	80.9	80.7	79.2	86.2

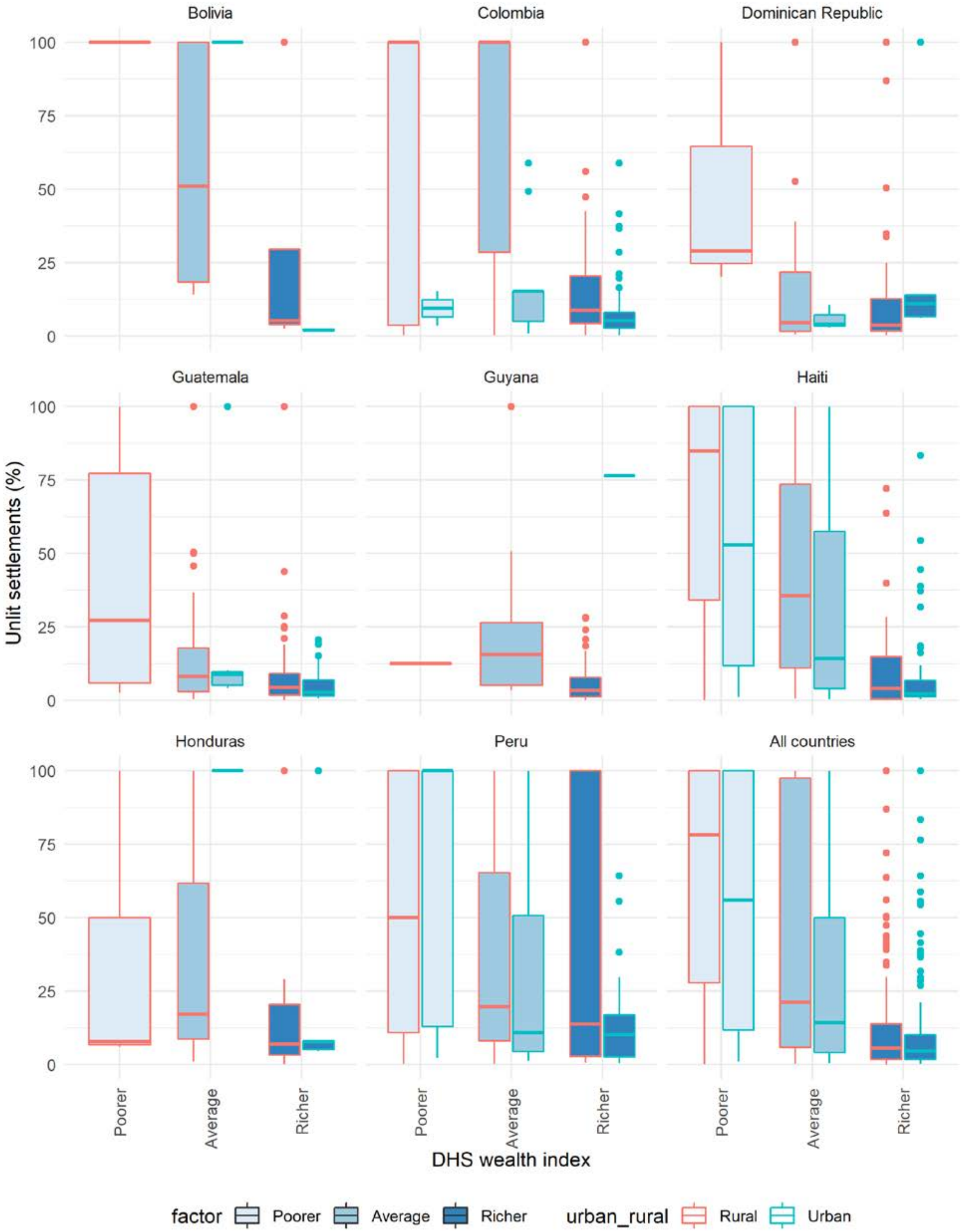
Bolivia
Colombia
Dominican Republic
Guatemala
Guyana
Haiti
Honduras
Peru
All countries
Unlit settlements (%)
100
75
50
25
0
Poorer
Average
Richer
DHS wealth index
factor
Poorer
Average
Richer
urban_rural
Rural
Urban

209

Supplementary Fig. 9. Boxplots for the Americas showing the mean percentage area of unlit settlements within a 2 km buffer (urban) or 5 km buffer (rural) of a DHS household cluster against the mode of the wealth indices of all households assigned to the household cluster. The midline represents the median with the lower and upper limits of the box being the 1st and 3rd quartiles.

Supplementary Table 8. Category-specific accuracy by country for the Americas.

	Poorer	Average	Richer	Overall
Bolivia	0	100	83.3	78.6
Colombia	31.7	83.8	87.3	82.3
Dominican Republic	66.7	99.3	88.9	94
Guatemala	68.8	89.9	94.6	91.6
Guyana	100	100	100	100
Haiti	45	90.6	76	77.8
Honduras	96.9	75.1	78	87.5
Peru	89.2	59.1	67.8	73.6
All	62.3	87.2	84.5	85.7

Supplementary Table 9. Overall continent-specific accuracy (for urban and rural settlements)

Continent	Rural Accuracy (%)	Urban Accuracy (%)
Africa	92.5	84.4
Asia	82.4	86.2
North America	82.2	83.6
South America	71.4	82.6

Supplementary References

1. Jean, N. *et al.* Combining satellite imagery and machine learning to predict poverty. *Science* **353**, 790 (2016).

2. Chen, X. & Nordhaus, W. D. Using luminosity data as a proxy for economic statistics. *Proc Natl Acad Sci USA* **108**, 8589 (2011).
3. Proville, J., Zavala-Araiza, D. & Wagner, G. Night-time lights: A global, long term look at links to socio-economic trends. *PLOS ONE* **12**, e0174610 (2017).
4. Doll, C. N. H., Muller, J.-P. & Morley, J. G. Mapping regional economic activity from night-time light satellite imagery. *Ecological Economics* **57**, 75–92 (2006).
5. Henderson, J. V., Storeygard, A. & Weil, D. N. Measuring Economic Growth from Outer Space. *American Economic Review* **102**, 994–1028 (2012).
6. Pinkovskiy, M. & Sala-i-Martin, X. Lights, Camera … Income! Illuminating the National Accounts-Household Surveys Debate *. *The Quarterly Journal of Economics* **131**, 579–631 (2016).
7. Kyba, C. C. M. *et al.* Artificially lit surface of Earth at night increasing in radiance and extent. *Sci Adv* **3**, (2017).
