# Supplementary material for: Estimating global economic well-being with unlit settlements
Source: Nat Commun. 2022 May 5;13:2459. doi: 10.1038/s41467-022-30099-9 (PMC9072384; doi:10.1038/s41467-022-30099-9)
Supplement: Supplementary file 2 — Description of Additional Supplementary Files [file 41467_2022_30099_MOESM2_ESM.pdf]

Description of Additional Supplementary files

File name: Supplementary Data 1

Description: National statistics of Total WSF settlement area (km2), Unlit WSF settlement area (km2), Total Unlit WSF (%), Urban Unlit (%) and Rural
